# Assessing the Mental Health Impact of the 2011 Great Japan Earthquake, Tsunami, and Radiation Disaster on Elementary and Middle School Children in the Fukushima Prefecture of Japan

**DOI:** 10.1371/journal.pone.0170402

**Published:** 2017-01-18

**Authors:** Mark Lieber

**Affiliations:** School of Medicine, University of California Irvine, Irvine, California, United States of America; Stellenbosch University, SOUTH AFRICA

## Abstract

**Background:**

On March 11, 2011, a magnitude 9.0 earthquake occurred off of Japan’s Pacific coast, which was followed by huge tsunamis that destroyed many coastal cities in the area. Due to the earthquake and subsequent tsunami, malfunctions occurred at the Fukushima Daiichi (Fukushima I) nuclear power plant, resulting in the release of radioactive material in the region. While recent studies have investigated the effects of these events on the mental health of adults in the region, no studies have yet been performed investigating similar effects among children.

**Methods and Findings:**

This study aims to fill that gap by: 1) assessing the mental health of elementary and middle school children living within the Fukushima prefecture of Japan, and 2) identifying risk and protective factors that are associated with the children’s mental health scores. These factors were quantified using an original demographics survey, the Strengths and Difficulties Questionnaire (SDQ), and the Impact of Event Scale–Revised (IES-R), the latter two of which have been previously validated in a Japanese setting. The surveys were distributed to approximately 3,650 elementary and middle school students during the months of February and March, 2012. The data suggests that those children who had been relocated to the city of Koriyama had significantly higher SDQ scores than those children who were native to Koriyama (p < .05) as well as a control group that lived outside of the Fukushima prefecture (p < .01). Using a multivariate regression, we also found that younger age and parental trauma were significantly correlated with higher SDQ scores (p < .001), while gender, displacement from one’s home, and exposure to violence were not.

**Conclusions:**

These results suggest that, among children affected by natural disasters, younger children and those with parents suffering from trauma-related distress are particularly vulnerable to the onset of pediatric mental disturbances.

## Introduction

On March 11, 2011, a 9.0 earthquake struck off the east coast of Tohoku, Japan, triggering a powerful tsunami that reached up to 40 meters high as well as a level 7 meltdown at three of the nuclear reactors in the Fukushima I Nuclear Power Plant complex. The earthquake was the strongest ever to hit Japan and the fifth largest ever to be recorded since seismological record keeping began in the late 1800’s. The earthquake, tsunami, and nuclear accident combined left more than 15,000 people dead, nearly 6,000 injured, and over 4,000 missing[1].

In the psychiatric literature, earthquakes have consistently been shown to be associated with mental health problems such as depression and post-traumatic stress disorder in the months and years immediately following the disaster. Approximately three years after a 1999 earthquake in Turkey, for example, the prevalence of comorbid depression among adults was found to be 18%, while the prevalence of post-traumatic stress disorder was 40%[2]. In the case of Fukushima, however, the presence of the tsunami as well as the threat of radiation poisoning is believed to have added to the mental health burden in the region[3].

### Mental Illness in Japan

Currently, over 80% of the mental health hospitals in Japan are private, and most are filled primarily with involuntary patients who are physically disabled, intellectually disabled, or who suffer from a severe psychotic disorder such as schizophrenia. As in many other countries, the mental healthcare system in Japan focuses on the long-term care of chronic disorders and developmental disabilities that exceed the caring capacity of families. Consequently, there are currently few hospitals or clinics in the country that people can visit to treat more acute disorders such as depression and post-traumatic stress disorder. While there has recently been a legislative push towards a more community-based mental healthcare system in Japan that focuses on these illnesses, the country’s mental health services today are still primarily reserved for the severely mentally ill[4].

In addition to the structural problems inherent in Japan’s mental healthcare system, many people in the country also refrain from using mental healthcare services due to the negative stigma attached to mental illness. According to Dr. Hiroshi Kato of the Hyogo Institute for Traumatic Stress in Kobe, *gaman*, or ‘endurance,’ is a very important attitude in Japanese culture that has caused many people in the country to respond to disasters with silence, avoidance, and patience[5]. Depression, which is often referred to by its euphemistic name, “heart flu,” is a particularly taboo subject in Japanese culture. Due to intense cultural pressures, individuals suffering from depression often end up feeling guilty or ashamed about discussing their psychological pain[6].

In addition to the stigma attached to depression, there is also a strong and deep-seeded stigma in Japan attached to radiation poisoning. More specifically, following the bombings of Hiroshima and Nagasaki during World War II, survivors of the disasters–referred to as “hibakusha”–were heavily discriminated against in other parts of the country. Today, individuals displaced from Fukushima have begun reporting similar types of discrimination, with some individuals being discouraged from seeking refuge in other prefectures for that reason. Children who have had to move to areas outside of the Fukushima prefecture have also begun reporting being bullied in elementary and middle schools due to their potential exposure to radiation[7].

### Pediatric Mental Illness in Japan

While the overall prevalence of pediatric mental illnesses in Japan is not known, a number of factors related to the March 11, 2011 disaster suggest that many of the children who were exposed to the earthquake, tsunami, and radiation scare are likely to suffer from moderate- to severe-levels of mental health problems such as depression and post-traumatic stress disorder.

The first such factor is the nature of the catastrophe itself. Previous studies have shown that children are often more susceptible than adults to mental health problems in the months and years following a natural disaster[8] and often manifest these problems through psychological withdrawal, temper tantrums, decreased concentration, and sleep disorders[9]. In addition to having to bear witness to the disaster itself, children are also known to worry more than adults about the possible recurrence of a disaster as well as the fate of family members, friends, and pets. Those who have already become separated from their parents often become confused about their own safety and the safety of their caregivers, while those who remain with their parents often worry that they may still become separated from them in the future. During a natural disaster, children are also known to be more vulnerable to stress than adults, particularly those who have witnessed people die or who have been trapped in damaged buildings or by debris for any length of time[10].

With regard to the radiation spill, the Japanese government’s decision to limit evacuation to within only 20 kilometers of the Fukushima Daiichi power plant has also been heavily criticized both domestically and internationally for its disproportionate impact on the region’s 300,000 children. This decision prompted six non-governmental organizations in Japan to submit a joint report to the UN Office of the High Commissioner for Human Rights (UNHCHR) describing the situation of the children in Fukushima and urging the United Nations to intervene[11]. Due to these factors, the need for more research investigating the prevalence of mental illnesses among the children of Fukushima as well as possible risk and protective factors is needed.

### Risk and Protective Factors for Pediatric Mental Illnesses

Three risk factors that appear to have a significant predictive effect on the onset of pediatric mental illnesses following a natural disaster include exposure to disaster-related violence, displacement from one’s home, and impaired parental mental health. Exposure to violence has repeatedly been shown to be a strong risk factor in the development of mental disorders among children affected by natural disasters. A study of Chilean refugees in Sweden indicated that rates of sleep disturbances and anxiety were higher among children who had witnessed violent events before migration[12]. Among Hurricane Katrina survivors, youths with a high exposure to violence were also more likely to develop long-term emotional disturbances than those with low- or moderate- exposure to violence[13].

Unlike exposure to violence, displacement from one’s home appears to have a more complicated predictive effect on the onset of pediatric mental illnesses. A number of studies focusing on Bosnian and Croatian refugees, for instance, have shown that while the prevalence of most mental illnesses does not differ significantly between displaced and non-displaced children, psychosocial adaptation remains worse in displaced populations[14]. Some of these studies have also suggested that displaced children have higher levels of anxiety and fewer coping strategies to deal with stressful situations than non-displaced children[15].

The existence of emotional and mental disturbances among the parents of children affected by a disaster has also been shown to be a significant risk factor in the development of pediatric mental illnesses. While researching the mental health of children affected by the 2003 earthquake in Iran, for instance, Kalantari and Vostanis found a significant positive relationship between children’s scores on the Strengths and Difficulties Questionnaire (SDQ) and their parents’ scores on the Self-Report Questionnaire (SRQ)[16].

In addition to these three risk factors, two factors that appear to be protective against the onset of pediatric mental illnesses include male gender and a younger age. More specifically, a number of studies have shown that depression is more prevalent among female refugee children than male refugee children[17]. Pyari’s research among survivors of the 2004 Indonesia tsunami[18] and Zhang’s research among survivors of the 2008 Sichuan earthquake in China[19] have also suggested that being male can be a strong protective factor against the onset of post-traumatic stress disorder. While the exact mechanisms behind this phenomenon are unclear, some researchers believe that the distinction can be attributed to differing coping mechanisms between girls and boys as well as more limited socio-economic resources for girls in many cultures[20].

Finally, numerous studies looking at the mental health of both children and adults have suggested that older survivors of natural disasters (>40 years) are more susceptible to mental health disorders such as post-traumatic stress disorder than their younger counterparts[21]. Within the youth population, however, very little is known about the protective effects of young age against mental illness.

Accordingly, we hypothesized that pediatric mental health outcomes, measured in sampled children using standardized Strengths and Difficulties questionnaires, would be influenced by exposure to violence, displacement from one’s home, impaired parental mental health, gender, and age.

## Methods

### Sample Size Calculations

The sample population was divided into three groups: students displaced from the coast to the city of Koriyama (category I–displaced children), students who were local to Koriyama (category II–non-displaced children), and students who lived outside of the Fukushima prefecture entirely (category III–controls). Using the Strengths and Difficulties Questionnaire in a Japanese population, the effect size often ranges from 2 to 3 with standard deviations ranging from 5 to 6[22]. With a two-sided alpha of 0.05 and beta of 0.20, the estimated number per group would therefore need to be between 45 and 176 in order to achieve a statistically significant effect.

### Exposure Variables

**Fig 1:**
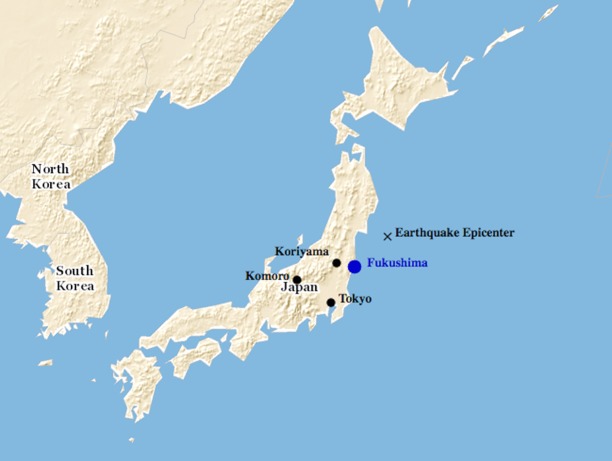
Map of Japan.

The students were divided into the three categories of displaced children, non-displaced children, and controls in order to distinguish the effects of the earthquake, relocation, and radiation spill (exposure variables) on the children’s mental health (outcome variable). Fig 1 shows the location of the two control cities in relation to Koriyama, the Fukushima power plant, and the earthquake’s epicenter. The epicenter of the earthquake was located approximately 80 km east of Japan’s coast, 200 km east of Koriyama, 375 km northeast of Komoro, and 400 km northeast of Tokyo.

According to the United States Geological Survey, “perceived shaking” was recorded as “very strong” to “severe” in Koriyama and along the eastern coast of the Fukushima prefectures, with peak ground velocities ranging from 16 to 70 centimeters/second. In Tokyo and Nagano, on the other hand, “perceived shaking” was recorded as “moderate” to “strong,” with peak ground velocities ranging from 3.4 to 16 centimeters/second[23]. Those children who were located in Koriyama and along the eastern coast were therefore exposed to much stronger tremors than those children who lived in the control areas of Nagano and Tokyo.

The radiation fallout in and around the Fukushima prefecture was also higher than in the control cities as measured by surface deposits of cesium-134 and -137. Internationally, a cesium fallout density of 10,000 becquerels per spare meter is recognized as the threshold for defining an area as being affected by a nuclear accident[24]. Koriyama was exposed to a combined cumulative density of between 60,000 and 100,000 becquerels per square meter. In Tokyo and Nagano, the fallout densities were considerably lower, measuring between 0 and 10,000 becquerels per square meter. Therefore, those children who lived in Koriyama and along the eastern coast of Fukushima were also exposed to considerably higher radiation levels than those children living in the control prefectures of Tokyo and Nagano.

### Participants

During the months of February and March, 2012, the researchers distributed 3,650 survey packets to nine schools across Japan. Of these, 3,000 were distributed to three elementary schools and four middle schools in Koriyama, Fukushima prefecture (categories I and II), 250 were distributed to one elementary school in Tachikawa, Tokyo prefecture (category III), and 400 were distributed to one middle school in Komoro, Nagano prefecture (category III). Because the researchers were unable to identify displaced children and their families in Koriyama using government records, surveys were distributed to all 3,000 children in the city with the hopes of receiving completed surveys from at least 45 displaced families–the threshold necessary to achieve a statistically significant effect.

By the end of April, the researchers had received a total of 1,004 completed surveys. Of these, 785 were returned from Koriyama (26%), 80 were returned from Tachikawa (32%), and 139 were returned from Komoro (35%). Of the 785 surveys returned from Koriyama, 51 were returned from displaced families and 734 were returned from native families. Table 1 shows the distribution of returned surveys by category.

**Table 1:** Sample Distribution by Category.

	Relocated (category I)	Native (category II)	Control (category III)	Total
Population (N)	51	734	219	1,004

### Ethics

Ethics approval for this study was obtained by the Institutional Review Board of the University of California—San Francisco and participant consent was implicit in the parent’s decision to return the completed survey to the researchers. The questionnaires were also approved by local Japanese school officials.

### Procedures

During the distribution of the survey packets in February and March, 2012, teachers in all nine participating schools were instructed to give the packets to their students during the last week of February and the first week of March. The students were then told by their teachers to give the packets to their parents upon returning home and to return the completed surveys via postal mail no later than the first week of April. Parents were provided with pre-addressed, stamped envelopes in order to successfully return the completed packets to the researchers. No other incentives to participate were employed.

The survey packets consisted of an original demographic survey, the Strengths and Difficulties Questionnaire, and the Impact of Event Scale-Revised, all of which were to be filled out by the children’s parents. The demographics survey was used to identify potential risk and protective factors, the Strengths and Difficulties Questionnaire (SDQ) was used to assess the mental health of the children (the main outcome variable), and the Impact of Event Scale-Revised (IES-R) was used to assess trauma in the children’s parents (one of the predictor variables). We decided upon these survey instruments over others that measure mental disorders due to the fact that they rely upon the responses of a child’s caregiver rather than the children themselves. Due to ethical and logistical constraints, we were not able to receive the necessary permission to evaluate the children directly. In addition to our research constraints, we also decided to rely upon the SDQ as our primary diagnostic tool because it is widely known for being one of the most reliable indicators of pediatric mental disturbances internationally, having been successfully used in Japan[25] as well as in other Asian countries such as Bangladesh, Pakistan, and Sri Lanka[26]. The SDQ includes 25 questions that are divided between five scales of five items each: Conduct Problems, Inattention-Hyperactivity, Emotional Symptoms, Peer Problems, and Prosocial Behaviors. These five subscores are then summed together to generate a Total Difficulties score of 0–40[27].

### Measures

**Aim 1:** After all of the survey packets had been returned to the researchers, they were then divided into the three categories outlined above: students displaced from the coast to the city of Koriyama (category I–displaced children), students who were local to Koriyama (category II–non-displaced children), and students who lived outside of the Fukushima prefecture entirely (category III–controls). The Strengths and Difficulties Questionnaires were then scored for all 1,004 children by determining each child’s Total Difficulties Score and comparing it to clinical range cut-offs first developed by Robert Goodman in the United Kingdom[28]. More specifically, SDQ Total Difficulties scores of 0–13 were classified as “normal,” 14–16 as “borderline,” and 17–40 as “abnormal.” Mean SDQ scores for each of the three groups were then compared to one another using two-sample t-tests.

**Aim 2:** In order to identify potential risk and protective factors, the researchers relied upon responses to the demographic survey as well as the results of the parents’ Impact of Event Scale-Revised (IES-R) questionnaires. The IES-R was used to assess subjective distress caused by the earthquake, tsunami, and radiation spill in the children’s parents. Respondents were asked to indicate how much they were distressed within the past seven days by a number of “difficulties” that correspond to 14 of the 17 DSM-IV symptoms of post-traumatic stress disorder (PTSD). Items were rated on a 5-point scale ranging from 0 (“not at all”) to 4 (“extremely”) and total scores out of 88 were used to assess the parents’ level of distress[29]. While IES-R scores and age were both treated as continuous variables, gender, exposure to violence, and displacement from one’s home were all treated as dichotomous variables. With regard to exposure to violence, questions 13 and 14 in the demographics survey asked parents to indicate whether the child was hurt during the earthquake, whether a family member or friend was injured or died during the earthquake, or whether the child saw someone get hurt or die during the earthquake. If the parent responded positively to any of these questions, the child was marked as having been exposed to violence. After assessing the potential risk and protective factors, the researchers then used a number of regression models to identify and isolate which risk and protective factors were significantly associated with the children’s SDQ Total Difficulties scores, taking potential confounders into effect.

## Results

**Aim 1:** The presence of a mental disorder was indicated by SDQ scores of 17 or greater as determined by SDQ developer Robert Goodman in 2011. Using these cut-off measures, the prevalence of mental disturbances was determined to be 20% among the relocated students (category I), 14% among the students native to Koriyama (category II), and 7% among the students outside of Fukushima (category III).

In addition to determining the prevalence of mental disturbances, the researchers also calculated the mean SDQ Total Difficulties score in the three sample populations. The mean Total Difficulties score among the relocated children was 17.2 (SD = 7.0); among the native students it was 15.6 (SD = 6.2); and among the controls it was 14.7 (SD = 5.4). Using a two-sample t-test, the mean score among the control group was found to be significantly lower than both the native group (p < .05) and the relocated group (p < .01).

**Fig 2:**
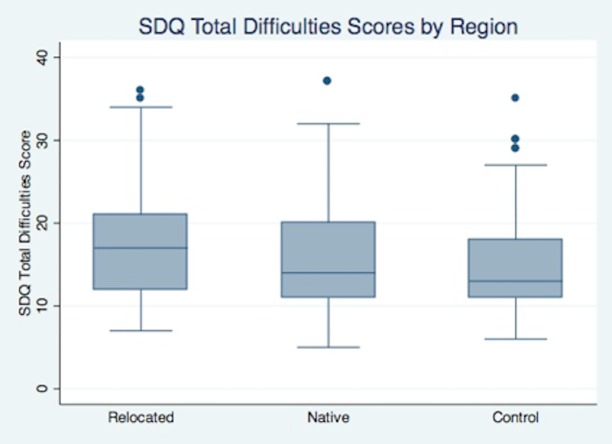
Box-Plot of SDQ Total Difficulties Scores by Category.

Fig 2 shows the distribution of SDQ scores within the relocated, native, and control groups. While all three categories included SDQ scores ranging from below 10 to above 30, the mean SDQ score among the relocated group (mean = 17.2) was significantly greater than the mean for either the native or control groups. In addition to the mean scores, the distribution of the SDQ Total Difficulties scores was also more tightly concentrated in the “normal” (0–13) and “borderline” (14–16) range among the control group (SD = 5.4) than among either the “relocated” or “native” groups.

Table 2 shows the mean and standard deviation of SDQ Total Difficulties scores by category, along with the percentage of each population that scored within the “abnormal” range of 17–40. Relocated children were found to have both a higher mean SDQ score and a greater prevalence of “abnormal” SDQ scores than children in the native and control groups. Those children who were native to Koriyama had mean SDQ scores and “abnormal” prevalence rates in between those of the other two groups.

**Table 2:** SDQ Total Difficulties Scores.

Category	Population (N)	Percentage with “abnormal” SDQ score	SDQ Total Difficulties Score (mean)	SDQ Score Standard Deviation	P-value
Relocated (category I)	51	20%	17.2	+/-7.0	p < .01**
Native (category II)	734	14%	15.6	+/-6.2	p < .05*
Control (category III)	219	7%	14.7	+/-5.4	-
Total	1,004	12%	15.6	+/-6.2	-

*—statistically significant.

**—very statistically significant.

Two-sample t-tests comparing the mean SDQ scores of the control group with those of the relocated group and the native group indicate that the differences in mean SDQ scores are statistically significant at a .05 significance level. This suggests that exposure to both the earthquake and radiation spill contributed to an increase in mental health problems within both the relocated and native student populations. Though not indicated in the table, a two-sample t-test comparing the mean SDQ scores of the relocated and native groups was also found to be statistically significant (p = .049), suggesting that the act of relocating may have also contributed to an increase in mental health problems within the relocated group. However, because the relocated children may have differed from the native children in other respects besides just relocation–including an increase in exposure to violence and loss of loved ones–it is difficult to make any definitive comparisons between these two groups without first controlling for other potential confounders.

**Aim 2:** After assessing the prevalence of mental disturbances among our three groups, the researchers then performed simple and multivariate regressions relating SDQ Total Difficulties scores to variables such as exposure to violence, displacement from one’s home, parental trauma, gender, and age. Table 3 shows the distribution of these variables by category. Unfortunately, due to constraints placed upon the researchers by the school boards of the Tokyo and Nagano prefectures, we were unable to distribute the IES-R questionnaire to the parents of control children. Therefore, parental trauma was only assessed as a potential risk factor among the relocated and native populations.

**Table 3:** Distribution of Risk and Protective Factors.

Category	Exposure to Violence	Displacement from Home	Average IESR Score	Male Gender	Average Age
Relocated (category I)	25%	100%	30.8	63%	11.6 years
Native (category II)	7%	0%	21.6	49%	12.2 years
Control (category III)	0%	0%	N/A	49%	12.2 years

In order to identify which risk and protective factors were significantly associated with children’s mental health status, the researchers first performed simple linear regressions using each of the five variables as predictors of SDQ Total Difficulties scores. Exposure to violence, displacement from one’s home, and gender were all treated as dichotomous variables while SDQ score, parental trauma, and age were treated as continuous variables.

**Fig 3:**
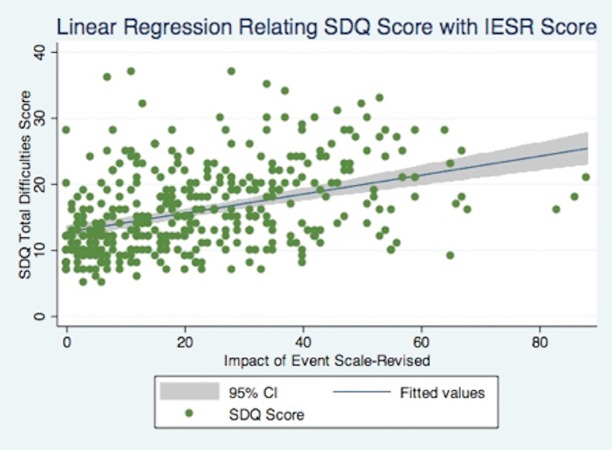
Simple Regression Relating SDQ Score with Parental Mental Health.

Of the five variables under investigation, four were found to have a statistically significant association with children’s SDQ Total Difficulties score using a simple linear regression: exposure to violence (β = 2.46, p = .011), displacement from one’s home (β = 2.22, p = .020), parental trauma (β = 0.144, p = .000), and age (β = -0.722, p = .000). The only factor that did not have a statistically significant association with SDQ score using a simple linear regression was gender (β = -0.032, p = .629). Fig 3 visualizes the results of the linear regression relating children’s SDQ scores with parents’ IES-R scores.

After completing the five simple linear regressions, the researchers then began looking at each of the four remaining variables in turn, using multivariate regressions to account for possible confounders.

### Exposure to Violence

Using a simple linear regression, exposure to violence appeared to be associated with children’s SDQ Total Difficulties scores (β = 2.46, p = .011). In order to account for any possible confounders, the investigators then performed a series of multivariate regressions to find out if this association remained while controlling for other potential factors.

First, the investigators looked at the relationship between exposure to violence and SDQ score while controlling for category type. Because the control children had not been exposed to disaster-related violence, the investigators looked at this correlation only within the native group and the relocated group. Among the native group, the correlation between these two variables remained significant (β = 2.41, p = .019) while, among the relocated children, there was no longer any significant correlation between SDQ score and exposure to violence (β = 2.16, p = .441).

In order to account for this discrepancy, the investigators then began looking for other possible confounders and found that, among the entire sample population, there was not a significant association between exposure to violence and SDQ score when controlling for parental trauma (β = 1.06, p = .247). When looking specifically at the native population, the correlation between exposure to violence and SDQ score also disappeared when controlling for parental trauma (β = 0.928, p = .339). Therefore, the investigators decided to discard exposure to violence as a potential risk factor for increased SDQ Total Difficulties scores.

### Displacement from Home

Like exposure to violence, displacement from one’s home also appeared to have a statistically significant association with children’s SDQ scores. Because only relocated children experienced this risk factor, the investigators decided to look at the other two remaining factors–parental trauma and age–as potential confounders. In doing so, the researchers discovered that while the association remained significant when controlling for age (β = 1.85, p = .043), it again disappeared when controlling for parental trauma (β = 0.914, p = .309). Therefore, displacement from one’s home was also discarded as a potential risk factor for increased SDQ Total Difficulties scores.

### Parental Trauma

Based on the simple linear regressions and the previous two multivariate regressions, parental trauma, as assessed by the Impact of Event Scale-Revised (IES-R), appeared to be a strong risk factor for impaired mental health among children. In order to confirm this, the investigators performed a series of multivariate regressions to control for age, displacement from one’s home, and exposure to violence. The association between parental trauma and children’s SDQ Total Difficulties scores remained statistically significant (p = .000) among all three analyses. Consequently, the researchers decided to keep parental trauma in their final regression model.

### Age

Finally, while the effects of age on mental health have not been adequately researched within the youth population, our simple regressions and multivariate models suggest that age may, in fact, be a significant risk factor in the development of mental disturbances among children affected by a natural disaster. In order to confirm this hypothesis, the investigators again performed a series of multivariate regressions, controlling for each of the other three potential risk factors. In all three models, age continued to be significantly associated with impaired mental health among children (p = .000). What was perhaps most interesting, though, was that this association was negative, meaning that younger children were more at risk for developing mental disturbances after a natural disaster than older children.

In sum, parental trauma (β = 0.128, p = .000, 95%CI .095-.161) and a younger age (β = -0.588, p = .000, 95%CI -.799- -.377) were the only two risk factors that were significantly associated with SDQ Total Difficulties score when adjusted for other factors. More specifically, each one-point increase in a child’s SDQ Total Difficulties score was associated with a .128-point increase in parental IES-R score and a 7-month decrease in age. Together, these two variables accounted for 21% of the variation in SDQ score. Gender, displacement from one’s home, and exposure to violence were not found to have a significant association with SDQ Total Difficulties scores.

## Discussion

In our study sample, the prevalence of mental disturbances was 7% among our control population, 14% among the native population, and 20% among the relocated population. While these figures are slightly lower than the 20–40% range indicated in previous studies, the 14% prevalence difference between the control and relocated groups suggests a real possible difference in the prevalence of mental illnesses between those children who were directly affected by the 2011 East Japan disaster and those who were living in other parts of Japan. This conclusion is also supported by the statistically significant difference in mean SDQ Total Difficulties scores between the three groups.

Within the Koriyama population, those children who had been relocated from the coast also had a significantly higher mean SDQ score than those children who were native to Koriyama. The difference in SDQ scores between the relocated and native groups suggests that, within the population of children affected by a natural disaster, relocated children are particularly vulnerable to developing mental disturbances one year after the event. However, in addition to having to relocate, these children were also more likely to lose their homes and loved ones in the tsunami, making it difficult to determine whether the relocation itself, or some other related factors, accounted for their higher SDQ scores.

With regard to risk factors, parental trauma and a younger age were the only two factors that were significantly associated with higher SDQ Total Difficulties scores. The fact that parental trauma was associated with higher SDQ scores suggests one of four things: that children tend to internalize and reflect trauma experienced by their parents, that parents who experience trauma are more likely to overestimate the existence of mental health problems in their children, that children prone to mental disturbances often come from parents who are more sensitive to traumatizing events, or that children and their parents who are exposed to similar levels of disaster-related stressors are more likely to experience mental health disturbances. While it is impossible to distinguish between these theories in our current study, future studies could attempt to do so either by measuring the children’s mental health directly or by using a third party, such as a teacher, to validate the parents’ evaluations of their children.

In addition to parental trauma, younger age was also a significant risk factor for developing mental disturbances after a natural disaster. In other words, children who were younger had, on average, higher SDQ Total Difficulties scores than their older counterparts. While this is inconsistent with previous studies that have shown an association between an increase in age and an increase in symptoms of post-traumatic stress disorder[30], it does tend to resonate with other research that has shown that younger children are more likely to worry about the possible recurrence of a disaster as well as the fate of family members, friends, and pets[31]. Regardless, this observation suggests that future, post-disaster mental health interventions might be more effective if they focus on a younger target population.

Surprisingly, factors such as gender, exposure to violence, and displacement from one’s home were not found to be significantly associated with children’s SDQ scores, as we had originally hypothesized. The fact that gender was not associated with SDQ score is inconsistent with previous studies that have shown a protective effect of male sex[32], though there have been a number of other studies that have also found no association between gender and the onset of mental disturbances among children[33]. The fact that exposure to violence and damage to one’s home were not statistically significant risk factors was also surprising, particularly given the fact that relocated children had, on average, higher SDQ scores than those children who were native to Koriyama.

Our study also has a number of limitations. The first is the low response rate (28%), which opens the study up to the potential for selection bias. More specifically, it is possible that those parents who decided to respond to our survey differed in some relevant factor, such as their mental health status, as compared to those parents who did not decide to respond. While there is no way for us to know if this is actually the case, the fact that the response rate was similar among all three groups—ranging from 26% in Koriyama to 35% in Komoro—suggests that the reasons underlying the low response rate were applicable to all groups. One reason for the low response rate may be that some parents did not receive the surveys due to a lack of distribution either within the schools (from the teachers to the students) or at home (from the students to the parents). In addition, our reliance on mail-in surveys may have presented an additional logistical barrier as previous research suggests that mail-in surveys typically result in response rates of 20–30%, which is consistent with the results of this study[34]. In order to increase the response rate in future studies, researchers should therefore aim to streamline the survey distribution and collection process, and avoid relying on mail-in surveys whenever possible. In addition to the low response rate, this study was also limited by our inability to prospectively identify relocated students. More specifically, because there was no government database of families who had been relocated to Koriyama from the coast, we had to resort to distributing the surveys to all elementary and middle school students in the city and hope that we received an adequate number of responses from relocated families. While this approach did provide us with an adequate sample size of relocated children (n = 51), it also prevented us from using stratified or random sampling techniques that would have helped mitigate the potential for selection bias. It is also possible that some of the students who relocated from the coast never enrolled in elementary or middle school in Koriyama and therefore never received the survey packet. Finally, the use of the SDQ as our primary metric tool has inherent limitations since it relies on the responses of the children’s parents rather than the children themselves. Consequently, bias on the part of the parents could have affected the study’s results, particularly since the parents were also exposed to the earthquake, tsunami, and radiation spill. While there is a self-report version of the SDQ for children aged 11–17, we did not use it due to ethical considerations as well as the fact that our sample included children as young as five years old.

The implications of our study can be divided into mitigating interventions that might be performed before, during, and after a natural disaster. Before a natural disaster, efforts should be taken to develop a more community-based model of mental healthcare delivery that can be relied upon during times of crisis. This is particularly relevant in Japan given the country’s current focus on hospital-based treatment of psychotic illnesses rather than a community-based model that addresses more prevalent illnesses such as depression, substance abuse, and post-traumatic stress disorder. During a disaster, the national government should also plan to mobilize and rely more heavily on public-health nurses and doctors from outside the prefecture that is being directly affected. While Japan did, in fact, develop a prefecture-based mental health response system after the 1995 Kobe earthquake, the system failed during the 2011 East Japan earthquake due to the fact that many public health workers living in the Fukushima prefecture were among the disaster’s victims[35]. Finally, with regard to post-disaster intervention strategies, our study suggests that disaster responders should focus more heavily on addressing the needs of very young children, who are more prone to developing mental disturbances in the wake of a disaster than their older counterparts. Particularly in countries like Japan where mental health is heavily stigmatized, identification of at-risk children may require coordination with school districts, with potential interventions ranging from play therapy to trauma-focused cognitive behavioral therapy (TFCBT)[36].

## Supporting Information

S1 Supporting Information (ZIP)
