# Supplementary material for: Assessing the Mental Health Impact of the 2011 Great Japan Earthquake, Tsunami, and Radiation Disaster on Elementary and Middle School Children in the Fukushima Prefecture of Japan
Source: PLoS One. 2017 Jan 18;12(1):e0170402. doi: 10.1371/journal.pone.0170402 (PMC5242473; doi:10.1371/journal.pone.0170402)
Supplement: S1 Supporting Information — (ZIP) [file pone.0170402.s001.zip › Capstone_Regression.doc]

___ ____ ____ ____ ____ (R)

/__	/	____/	/	____/

___/	/	/___/	/	/___/	12.0	Copyright 1985-2011 StataCorp LP

Statistics/Data Analysis	StataCorp

4905 Lakeway Drive

College Station, Texas 77845 USA

800-STATA-PC	http://www.stata.com

979-696-4600	stata@stata.com

979-696-4601 (fax)

Single-user Stata license expires 13 Sep 2012:

Serial number: 30120932924

Licensed to: Mark Lieber

UCSF

LINEAR REGRESION BETWEEN SQD TOTAL DIFFICULTIES SCORE AND PARENT’S IESR SCORE – STATISTICALLY SIGNIFICANT

. spearman TotalDiff IESR

Number of obs =	380

Spearman's rho =	0.4297

Test of Ho: TotalDiff and IESR are independent

Prob > |t| =	0.0000

# LOGISTICAL REGRESSION USING SDQ AND IESR SCORES – STATISTICALLY SIGNIFICANT

. regress TotalDiff IESR

Source |	SS	df	MS	Number of obs =	380

-------------+------------------------------	F( 1, 378) =	67.61

Model | 2394.98101	1 2394.98101	Prob > F	= 0.0000

Residual |	13389.24	378	35.42127	R-squared	= 0.1517

-------------+------------------------------	Adj R-squared = 0.1495

Total | 15784.2211	379 41.6470212	Root MSE	= 5.9516

------------------------------------------------------------------------------

TotalDiff |	Coef. Std. Err. t	P>|t|	[95% Conf. Interval]

-------------+----------------------------------------------------------------

IESR |	.1436822	.0174737	8.22	0.000	.1093244	.17804

_cons |	12.7781	.5033204	25.39	0.000	11.78844	13.76776

------------------------------------------------------------------------------

# LOGISTICAL REGRESSION USING IESR SCORE AND AGE AS PREDICTORS FOR SDQ SCORE – BOTH STATISTICALLY SIGNIFICANT

. regress TotalDiff IESR Age

Source |	SS	df	MS	Number of obs =	380

-------------+------------------------------	F( 2, 377) =	51.41

Model | 3382.52336	2 1691.26168	Prob > F	= 0.0000

Residual | 12401.6977	377 32.8957498	R-squared	= 0.2143

-------------+------------------------------	Adj R-squared = 0.2101

Total | 15784.2211	379 41.6470212	Root MSE	= 5.7355

------------------------------------------------------------------------------

TotalDiff |	Coef. Std. Err. t	P>|t|	[95% Conf. Interval]

-------------+----------------------------------------------------------------

IESR |	.1281535	.0170761	7.50	0.000	.0945772	.1617297

Age | -.5878761	.1072946	-5.48	0.000	-.798847	-.3769053

_cons |	20.25938	1.44902	13.98	0.000	17.41021	23.10856

------------------------------------------------------------------------------

LOGISTICAL REGRESSION USING SCHOOL TYPE AS PREDICTOR FOR SDQ SCORE – STATISTICALLY SIGNIFICANT

. regress TotalDiff School

Source |	SS	df	MS	Number of obs =	380

-------------+------------------------------	F( 1, 378) =	18.17

Model | 724.019667	1 724.019667	Prob > F	= 0.0000

Residual | 15060.2014	378 39.8418026	R-squared	= 0.0459

-------------+------------------------------	Adj R-squared = 0.0433

Total | 15784.2211	379 41.6470212	Root MSE	=	6.312

------------------------------------------------------------------------------

TotalDiff |	Coef. Std. Err. t	P>|t|	[95% Conf. Interval]

-------------+----------------------------------------------------------------

School | -2.950176	.6920575	-4.26	0.000	-4.31094	-1.589411

_cons |	17.02335	.393734	43.24	0.000	16.24916	17.79753

------------------------------------------------------------------------------

LOGISTICAL REGRESSION USING GENDER AS PREDICTOR OF SDQ SCORE – NOT STATISTICALLY SIGNIFICANT

. regress TotalDiff Gender

Source |	SS	df	MS	Number of obs =	380

-------------+------------------------------	F( 1, 378) =	0.23

Model | 9.73526069	1 9.73526069	Prob > F	= 0.6294

Residual | 15774.4858	378 41.7314439	R-squared	= 0.0006

-------------+------------------------------	Adj R-squared = -0.0020

Total | 15784.2211	379 41.6470212	Root MSE	=	6.46

------------------------------------------------------------------------------

TotalDiff |	Coef. Std. Err. t	P>|t|	[95% Conf. Interval]

-------------+----------------------------------------------------------------

Gender | -.0318809	.0660068	-0.48	0.629	-.1616675	.0979056

_cons |	16.12404	.3508311	45.96	0.000	15.43422	16.81387

------------------------------------------------------------------------------

LOGISTICAL REGRESSION USING EXPOSURE TO VIOLENCE AS PREDICTOR FOR SDQ SCORE – STATISTICALLY SIGNICANT

. regress TotalDiff ExpViol

Source |	SS	df	MS	Number of obs =	380

-------------+------------------------------	F( 1, 378) =	6.50

Model |	266.66208	1	266.66208	Prob > F	= 0.0112

Residual |	15517.559	378 41.0517433	R-squared	= 0.0169

-------------+------------------------------	Adj R-squared = 0.0143

Total | 15784.2211	379 41.6470212	Root MSE	= 6.4072

------------------------------------------------------------------------------

TotalDiff |	Coef. Std. Err. t	P>|t|	[95% Conf. Interval]

-------------+----------------------------------------------------------------

ExpViol |	2.457477	.9642165	2.55	0.011	.5615766	4.353377

_cons |	15.7386	.3532384	44.56	0.000	15.04404	16.43316

------------------------------------------------------------------------------

LOGISTICAL REGRESSION USING DAMAGE TO HOME AS PREDICTOR FOR SDQ SCORE – STATISTICALLY SIGNIFICANT

. regress TotalDiff Damag

Source |	SS	df	MS	Number of obs =	380

-------------+------------------------------	F( 1, 378) =	7.37

Model | 301.676225	1 301.676225	Prob > F	= 0.0070

Residual | 15482.5448	378 40.9591133	R-squared	= 0.0191

-------------+------------------------------	Adj R-squared = 0.0165

Total | 15784.2211	379 41.6470212	Root MSE	= 6.3999

------------------------------------------------------------------------------

TotalDiff |	Coef. Std. Err. t	P>|t|	[95% Conf. Interval]

-------------+----------------------------------------------------------------

Damag |	.0364558	.013433	2.71	0.007	.0100431	.0628685

_cons |	15.8162	.3412096	46.35	0.000	15.1453	16.48711

------------------------------------------------------------------------------

LOGISTICAL REGRESSION USING IESR, AGE, SCHOOL TYPE, EXPOSURE TO VIOLENCE, AND DAMAGE TO HOME AS PREDICTORS OF SDQ SCORES. IESR, AGE, AND DAMAGE TO HOME STILL STATISTICALLY SIGNIFICANT. SCHOOL TYPE AND EXPOSURE TO VIOLENCE NOT STATISTICALLY SIGNIFICANT. ACCOUNTS FOR 23% OF VARIABILITY

. regress TotalDiff IESR Age School ExpViol Damag

Source |	SS	df	MS	Number of obs =	380

-------------+------------------------------	F( 5, 374) =	22.62

Model | 3665.16633	5 733.033266	Prob > F	= 0.0000

Residual | 12119.0547	374 32.4038896	R-squared	= 0.2322

-------------+------------------------------	Adj R-squared = 0.2219

Total | 15784.2211	379 41.6470212	Root MSE	= 5.6924

------------------------------------------------------------------------------

TotalDiff |	Coef. Std. Err. t	P>|t|	[95% Conf. Interval]

-------------+----------------------------------------------------------------

IESR |	.1231907	.0173719	7.09	0.000	.0890319	.1573495

Age | -.7643046	.1686896	-4.53	0.000	-1.096004	-.4326056

School |	1.435059	1.00316	1.43	0.153	-.537482	3.4076

ExpViol |	1.138399	.8753923	1.30	0.194	-.582909	2.859706

Damag |	.026582	.0120166	2.21	0.028	.0029534	.0502106

_cons |	21.71033	1.900654	11.42	0.000	17.97302	25.44764

. regress TotalDiff IESR Age School ExpViol Damag Displacement

Source |	SS	df	MS	Number of obs =	356

-------------+------------------------------	F( 6, 349) =	18.01

Model | 3293.08105	6 548.846842	Prob > F	= 0.0000

Residual | 10637.4667	349 30.4798473	R-squared	= 0.2364

-------------+------------------------------	Adj R-squared = 0.2233

Total | 13930.5478	355 39.2409796	Root MSE	= 5.5209

------------------------------------------------------------------------------

TotalDiff |	Coef. Std. Err. t	P>|t|	[95% Conf. Interval]

-------------+----------------------------------------------------------------

IESR |	.1333242	.0184728	7.22	0.000	.0969921	.1696563

Age | -.7736672	.1705746	-4.54	0.000	-1.109151	-.4381837

School |	1.532589	1.019866	1.50	0.134	-.4732672	3.538446

ExpViol |	.9728017	.8688932	1.12	0.264	-.736124	2.681727

Damag | -.0092262	.055933	-0.16	0.869	-.1192344	.100782

Displacement |	.5855244	.8893346	0.66	0.511	-1.163605	2.334654

_cons |	21.52313	1.912883	11.25	0.000	17.7609	25.28536

. regress TotalDiff IESR Age School ExpViol Damag

Source |	SS	df	MS	Number of obs =	356

-------------+------------------------------	F( 5, 350) =	21.56

Model | 3279.86893	5 655.973786	Prob > F	= 0.0000

Residual | 10650.6788	350 30.4305109	R-squared	= 0.2354

-------------+------------------------------	Adj R-squared = 0.2245

Total | 13930.5478	355 39.2409796	Root MSE	= 5.5164

------------------------------------------------------------------------------

TotalDiff |	Coef. Std. Err. t	P>|t|	[95% Conf. Interval]

-------------+----------------------------------------------------------------

IESR |	.1356566	.0181153	7.49	0.000	.1000281	.1712851

Age |	-.759998	.1691693	-4.49	0.000	-1.092714	-.4272818

School |	1.416894	1.003799	1.41	0.159	-.5573438	3.391131

ExpViol |	.970663	.8681836	1.12	0.264	-.7368502	2.678176

Damag | -.0087776	.0558836	-0.16	0.875	-.1186875	.1011322

_cons |	21.42084	1.90502	11.24	0.000	17.67411	25.16756

------------------------------------------------------------------------------

MHODDS/REGRESSION USING BINARY AND ALL FUKUSHIMA/RELOCATED

. mhodds totaldiffbin iesr

Score test for trend of odds with iesr

(The Odds Ratio estimate is an approximation to the odds ratio

for a one unit increase in iesr)

----------------------------------------------------------------

Odds Ratio	chi2(1)	P>chi2	[95% Conf. Interval]

----------------------------------------------------------------

1.042222	47.95	0.0000	1.030094	1.054494

----------------------------------------------------------------

. mhodds totaldiffbin age

Score test for trend of odds with age

(The Odds Ratio estimate is an approximation to the odds ratio

for a one unit increase in age)

----------------------------------------------------------------

Odds Ratio	chi2(1)	P>chi2	[95% Conf. Interval]

----------------------------------------------------------------

0.811132	31.11	0.0000	0.753614	0.873040

----------------------------------------------------------------

. mhodds totaldiffbin expviol

Maximum likelihood estimate of the odds ratio

Comparing expviol==1 vs. expviol==0

----------------------------------------------------------------

Odds Ratio	chi2(1)	P>chi2	[95% Conf. Interval]

----------------------------------------------------------------

2.151273	6.51	0.0108	1.176897	3.932353

----------------------------------------------------------------

. mhodds totaldiffbin damag

Maximum likelihood estimate of the odds ratio

Comparing damag==1 vs. damag==0

----------------------------------------------------------------

Odds Ratio	chi2(1)	P>chi2	[95% Conf. Interval]

----------------------------------------------------------------

1.525822	3.73	0.0535	0.990432	2.350623

----------------------------------------------------------------

. mhodds totaldiffbin displacement

Maximum likelihood estimate of the odds ratio

Comparing displacement==1 vs. displacement==0

----------------------------------------------------------------

Odds Ratio	chi2(1)	P>chi2	[95% Conf. Interval]

----------------------------------------------------------------

1.952667	5.13	0.0235	1.082611	3.521954

----------------------------------------------------------------

. mhodds totaldiffbin gender

Score test for trend of odds with gender

(The Odds Ratio estimate is an approximation to the odds ratio

for a one unit increase in gender)

----------------------------------------------------------------

Odds Ratio	chi2(1)	P>chi2	[95% Conf. Interval]

----------------------------------------------------------------

0.982575	0.72	0.3977	0.943351	1.023430

----------------------------------------------------------------

. regress totaldiffbin iesr

Source |	SS	df	MS	Number of obs =	380

-------------+------------------------------	F( 1, 378) =	54.75

Model | 11.5869842	1 11.5869842	Prob > F	= 0.0000

Residual | 80.0024894	378 .211646797	R-squared	= 0.1265

-------------+------------------------------	Adj R-squared = 0.1242

Total | 91.5894737	379	.24166088	Root MSE	= .46005

------------------------------------------------------------------------------

totaldiffbin |	Coef. Std. Err. t	P>|t|	[95% Conf. Interval]

-------------+----------------------------------------------------------------

iesr |	.0099939	.0013507	7.40	0.000	.0073381	.0126498

_cons |	.1764017	.0389062	4.53	0.000	.0999021	.2529013

------------------------------------------------------------------------------

. regress totaldiffbin age

Source |	SS	df	MS	Number of obs =	380

-------------+------------------------------	F( 1, 378) =	33.81

Model | 7.51913899	1 7.51913899	Prob > F	= 0.0000

Residual | 84.0703347	378 .222408293	R-squared	= 0.0821

-------------+------------------------------	Adj R-squared = 0.0797

Total | 91.5894737	379	.24166088	Root MSE	=	.4716

------------------------------------------------------------------------------

totaldiffbin |	Coef. Std. Err. t	P>|t|	[95% Conf. Interval]

-------------+----------------------------------------------------------------

age | -.0505855	.0087	-5.81	0.000	-.0676919	-.0334791

_cons |	1.018413	.1081923	9.41	0.000	.8056788	1.231147

------------------------------------------------------------------------------

. regress totaldiffbin expviol

Source |	SS	df	MS	Number of obs =	380

-------------+------------------------------	F( 1, 378) =	6.60

Model | 1.57207098	1 1.57207098	Prob > F	= 0.0106

Residual | 90.0174027	378 .238141277	R-squared	= 0.0172

-------------+------------------------------	Adj R-squared = 0.0146

Total | 91.5894737	379	.24166088	Root MSE	=	.488

------------------------------------------------------------------------------

totaldiffbin |	Coef. Std. Err. t	P>|t|	[95% Conf. Interval]

-------------+----------------------------------------------------------------

expviol |	.1886882	.0734389	2.57	0.011	.0442883	.3330882

_cons |	.3799392	.0269042	14.12	0.000	.3270387	.4328398

------------------------------------------------------------------------------

. regress totaldiffbin displacement

Source |	SS	df	MS	Number of obs =	380

-------------+------------------------------	F( 1, 378) =	5.19

Model | 1.24027283	1 1.24027283	Prob > F	= 0.0233

Residual | 90.3492009	378	.23901905	R-squared	= 0.0135

-------------+------------------------------	Adj R-squared = 0.0109

Total | 91.5894737	379	.24166088	Root MSE	=	.4889

------------------------------------------------------------------------------

totaldiffbin |	Coef. Std. Err. t	P>|t|	[95% Conf. Interval]

-------------+----------------------------------------------------------------

displacement |	.1649068	.0723929	2.28	0.023	.0225635	.3072501

_cons |	.382263	.027036	14.14	0.000	.3291032	.4354228

------------------------------------------------------------------------------

. regress totaldiffbin damag

Source |	SS	df	MS	Number of obs =	355

-------------+------------------------------	F( 1, 353) =	3.76

Model | .894966907	1 .894966907	Prob > F	= 0.0534

Residual | 84.1022162	353 .238249904	R-squared	= 0.0105

-------------+------------------------------	Adj R-squared = 0.0077

Total | 84.9971831	354 .240105037	Root MSE	= .48811

------------------------------------------------------------------------------

totaldiffbin |	Coef. Std. Err. t	P>|t|	[95% Conf. Interval]

-------------+----------------------------------------------------------------

damag |	.1013116	.0522724	1.94	0.053	-.0014928	.2041161

_cons |	.3532338	.0344285	10.26	0.000	.285523	.4209446

------------------------------------------------------------------------------

. regress totaldiffbin iesr age

Source |	SS	df	MS	Number of obs =	380

-------------+------------------------------	F( 2, 377) =	41.30

Model | 16.4611746	2 8.23058732	Prob > F	= 0.0000

Residual | 75.1282991	377 .199279308	R-squared	= 0.1797

-------------+------------------------------	Adj R-squared = 0.1754

Total | 91.5894737	379	.24166088	Root MSE	= .44641

------------------------------------------------------------------------------

totaldiffbin |	Coef. Std. Err. t	P>|t|	[95% Conf. Interval]

-------------+----------------------------------------------------------------

iesr |	.008903	.0013291	6.70	0.000	.0062897	.0115163

age | -.0413009	.008351	-4.95	0.000	-.0577213	-.0248805

_cons |	.7019947	.1127809	6.22	0.000	.4802364	.9237531

------------------------------------------------------------------------------

. regress totaldiffbin iesr age expviol

Source |	SS	df	MS	Number of obs =	380

-------------+------------------------------	F( 3, 376) =	28.22

Model | 16.8332915	3 5.61109718	Prob > F	= 0.0000

Residual | 74.7561822	376 .198819633	R-squared	= 0.1838

-------------+------------------------------	Adj R-squared = 0.1773

Total | 91.5894737	379	.24166088	Root MSE	= .44589

------------------------------------------------------------------------------

totaldiffbin |	Coef. Std. Err. t	P>|t|	[95% Conf. Interval]

-------------+----------------------------------------------------------------

iesr |	.008545	.0013531	6.32	0.000	.0058844	.0112055

age | -.0413502	.0083415	-4.96	0.000	-.0577519	-.0249484

expviol |	.0936052	.0684211	1.37	0.172	-.0409307	.2281412

_cons |	.6982279	.1126844	6.20	0.000	.4766574	.9197984

------------------------------------------------------------------------------

. mhodds totaldiffbin iesr age

Score test for trend of odds with iesr

controlling for age

note: only 10 of the 14 strata formed in this analysis contribute

information about the effect of the explanatory variable

(The Odds Ratio estimate is an approximation to the odds ratio

for a one unit increase in iesr)

----------------------------------------------------------------

Odds Ratio	chi2(1)	P>chi2	[95% Conf. Interval]

----------------------------------------------------------------

1.038048	34.54	0.0000	1.025200	1.051056

----------------------------------------------------------------

. mhodds totaldiffbin age iesr

Score test for trend of odds with age

controlling for iesr

note: only 46 of the 67 strata formed in this analysis contribute

information about the effect of the explanatory variable

(The Odds Ratio estimate is an approximation to the odds ratio

for a one unit increase in age)

----------------------------------------------------------------

Odds Ratio	chi2(1)	P>chi2	[95% Conf. Interval]

----------------------------------------------------------------

0.821866	19.74	0.0000	0.753728	0.896164

----------------------------------------------------------------

. mhodds totaldiffbin expviol

Maximum likelihood estimate of the odds ratio

Comparing expviol==1 vs. expviol==0

----------------------------------------------------------------

Odds Ratio	chi2(1)	P>chi2	[95% Conf. Interval]

----------------------------------------------------------------

2.151273	6.51	0.0108	1.176897	3.932353

----------------------------------------------------------------

. mhodds totaldiff expviol age

Response totaldiff not coded 0/1

r(198);

. mhodds totaldiffbin expviol age

Mantel-Haenszel estimate of the odds ratio

Comparing expviol==1 vs. expviol==0, controlling for age

note: only 9 of the 14 strata formed in this analysis contribute

information about the effect of the explanatory variable

----------------------------------------------------------------

Odds Ratio	chi2(1)	P>chi2	[95% Conf. Interval]

----------------------------------------------------------------

2.093202	4.86	0.0275	1.069049	4.098498

----------------------------------------------------------------

. mhodds totaldiffbin expviol iesr

Mantel-Haenszel estimate of the odds ratio

Comparing expviol==1 vs. expviol==0, controlling for iesr

note: only 25 of the 67 strata formed in this analysis contribute

information about the effect of the explanatory variable

----------------------------------------------------------------

Odds Ratio	chi2(1)	P>chi2	[95% Conf. Interval]

----------------------------------------------------------------

1.127005	0.09	0.7625	0.518757	2.448429

----------------------------------------------------------------

REGRESSIONS USING JUST MIDDLE SCHOOL (FUKUSHIMA/RELOCATED)

. regress totaldiff iesr

Source |	SS	df	MS	Number of obs =	123

-------------+------------------------------	F( 1, 121) =	22.99

Model | 653.818946	1 653.818946	Prob > F	= 0.0000

Residual | 3440.52252	121 28.4340704	R-squared	= 0.1597

-------------+------------------------------	Adj R-squared = 0.1527

Total | 4094.34146	122 33.5601759	Root MSE	= 5.3324

------------------------------------------------------------------------------

totaldiff |	Coef. Std. Err. t	P>|t|	[95% Conf. Interval]

-------------+----------------------------------------------------------------

iesr |	.1498813	.0312564	4.80	0.000	.0880011	.2117615

_cons |	11.2961	.7527052	15.01	0.000	9.805923	12.78628

------------------------------------------------------------------------------

. regress totaldiff expviol

Source |	SS	df	MS	Number of obs =	123

-------------+------------------------------	F( 1, 121) =	0.00

Model |	.09847276	1	.09847276	Prob > F	= 0.9571

Residual | 4094.24299	121 33.8367189	R-squared	= 0.0000

-------------+------------------------------	Adj R-squared = -0.0082

Total | 4094.34146	122 33.5601759	Root MSE	= 5.8169

------------------------------------------------------------------------------

totaldiff |	Coef. Std. Err. t	P>|t|	[95% Conf. Interval]

-------------+----------------------------------------------------------------

expviol | -.0841121	1.559175	-0.05	0.957	-3.17091	3.002686

_cons |	14.08411	.5623442	25.05	0.000	12.9708	15.19742

------------------------------------------------------------------------------

. regress totaldiff damag

Source |	SS	df	MS	Number of obs =	116

-------------+------------------------------	F( 1, 114) =	0.02

Model | .557586207	1 .557586207	Prob > F	= 0.8942

Residual |	3578.02	114 31.3861404	R-squared	= 0.0002

-------------+------------------------------	Adj R-squared = -0.0086

Total | 3578.57759	115	31.118066	Root MSE	= 5.6023

------------------------------------------------------------------------------

totaldiff |	Coef. Std. Err. t	P>|t|	[95% Conf. Interval]

-------------+----------------------------------------------------------------

damag |	-.14	1.050367	-0.13	0.894	-2.220769	1.940769

_cons |	14	.6895996	20.30	0.000	12.63391	15.36609

------------------------------------------------------------------------------

. regress totaldiff displacement

Source |	SS	df	MS	Number of obs =	123

-------------+------------------------------	F( 1, 121) =	0.25

Model | 8.49294125	1 8.49294125	Prob > F	= 0.6169

Residual | 4085.84852	121 33.7673432	R-squared	= 0.0021

-------------+------------------------------	Adj R-squared = -0.0062

Total | 4094.34146	122 33.5601759	Root MSE	=	5.811

------------------------------------------------------------------------------

totaldiff |	Coef. Std. Err. t	P>|t|	[95% Conf. Interval]

-------------+----------------------------------------------------------------

displacement |	1.134236	2.261637	0.50	0.617	-3.343271	5.611744

_cons |	14.00862	.5395348	25.96	0.000	12.94047	15.07677

REGRESSION USING JUST ELEMENTARY (FUKUSHIMA/RELOCATED)

. regress totaldiff iesr

Source |	SS	df	MS	Number of obs =	257

-------------+------------------------------	F( 1, 255) =	36.63

Model | 1377.43525	1 1377.43525	Prob > F	= 0.0000

Residual | 9588.42468	255 37.6016654	R-squared	= 0.1256

-------------+------------------------------	Adj R-squared = 0.1222

Total | 10965.8599	256 42.8353903	Root MSE	=	6.132

------------------------------------------------------------------------------

totaldiff |	Coef. Std. Err. t	P>|t|	[95% Conf. Interval]

-------------+----------------------------------------------------------------

iesr |	.1284915	.0212296	6.05	0.000	.0866838	.1702993

_cons |	13.81206	.6540798	21.12	0.000	12.52397	15.10014

------------------------------------------------------------------------------

. regress totaldiff gender

Source |	SS	df	MS	Number of obs =	257

-------------+------------------------------	F( 1, 255) =	0.44

Model | 18.9572205	1 18.9572205	Prob > F	= 0.5070

Residual | 10946.9027	255 42.9290302	R-squared	= 0.0017

-------------+------------------------------	Adj R-squared = -0.0022

Total | 10965.8599	256 42.8353903	Root MSE	=	6.552

------------------------------------------------------------------------------

totaldiff |	Coef. Std. Err. t	P>|t|	[95% Conf. Interval]

-------------+----------------------------------------------------------------

gender |	-.044586	.0670945	-0.66	0.507	-.176716	.0875439

_cons |	17.10645	.4274072	40.02	0.000	16.26475	17.94814

------------------------------------------------------------------------------

. regress totaldiff damag

Source |	SS	df	MS	Number of obs =	239

-------------+------------------------------	F( 1, 237) =	1.97

Model | 80.2806593	1 80.2806593	Prob > F	= 0.1616

Residual | 9650.15449	237 40.7179514	R-squared	= 0.0083

-------------+------------------------------	Adj R-squared = 0.0041

Total | 9730.43515	238 40.8841813	Root MSE	= 6.3811

------------------------------------------------------------------------------

totaldiff |	Coef. Std. Err. t	P>|t|	[95% Conf. Interval]

-------------+----------------------------------------------------------------

damag |	1.169017	.8325462	1.40	0.162	-.471119	2.809153

_cons |	16.24444	.5491944	29.58	0.000	15.16252	17.32637

------------------------------------------------------------------------------

. regress totaldiff expviol

Source |	SS	df	MS	Number of obs =	257

-------------+------------------------------	F( 1, 255) =	9.33

Model | 387.105353	1 387.105353	Prob > F	= 0.0025

Residual | 10578.7546	255	41.485312	R-squared	= 0.0353

-------------+------------------------------	Adj R-squared = 0.0315

Total | 10965.8599	256 42.8353903	Root MSE	= 6.4409

------------------------------------------------------------------------------

totaldiff |	Coef. Std. Err. t	P>|t|	[95% Conf. Interval]

-------------+----------------------------------------------------------------

expviol |	3.57825	1.171395	3.05	0.002	1.27141	5.88509

_cons |	16.53604	.4322855	38.25	0.000	15.68473	17.38734

------------------------------------------------------------------------------

. regress totaldiff displacement

Source |	SS	df	MS	Number of obs =	257

-------------+------------------------------	F( 1, 255) =	2.54

Model | 108.205379	1 108.205379	Prob > F	= 0.1121

Residual | 10857.6545	255 42.5790374	R-squared	= 0.0099

-------------+------------------------------	Adj R-squared = 0.0060

Total | 10965.8599	256 42.8353903	Root MSE	= 6.5253

------------------------------------------------------------------------------

totaldiff |	Coef. Std. Err. t	P>|t|	[95% Conf. Interval]

-------------+----------------------------------------------------------------

displacement |	1.692664	1.061804	1.59	0.112	-.3983574	3.783686

_cons |	16.72038	.4492175	37.22	0.000	15.83573	17.60503

------------------------------------------------------------------------------

. regress totaldiff iesr expviol

Source |	SS	df	MS	Number of obs =	257

-------------+------------------------------	F( 2, 254) =	20.26

Model | 1508.95551	2 754.477754	Prob > F	= 0.0000

Residual | 9456.90441	254 37.2319071	R-squared	= 0.1376

-------------+------------------------------	Adj R-squared = 0.1308

Total | 10965.8599	256 42.8353903	Root MSE	= 6.1018

------------------------------------------------------------------------------

totaldiff |	Coef. Std. Err. t	P>|t|	[95% Conf. Interval]

-------------+----------------------------------------------------------------

iesr |	.1191355	.0217036	5.49	0.000	.0763936	.1618774

expviol |	2.142828	1.140115	1.88	0.061	-.1024545	4.38811

_cons |	13.75406	.651587	21.11	0.000	12.47086	15.03726

----------------------------------------------------------------------------

# RELOCATED

summarize totaldiff

Variable |	Obs	Mean	Std. Dev. Min	Max

-------------+--------------------------------------------------------

totaldiff |	50	17.22	6.987715	7	35

# FUKUSHIMA – TOTAL

. summarize totaldiff

Variable |	Obs	Mean	Std. Dev. Min	Max

-------------+--------------------------------------------------------

totaldiff |	322	15.63665	6.165043	5	37

# FUKUSHIMA - ELEMENTARY

. summarize totaldiff

Variable |	Obs	Mean	Std. Dev. Min	Max

-------------+--------------------------------------------------------

totaldiff |	208	16.64904	6.347954	5	37

# FUKUSHIMA – MIDDLE

. summarize totaldiff

Variable |	Obs	Mean	Std. Dev. Min	Max

-------------+--------------------------------------------------------

totaldiff |	114	13.78947	5.368663	5	37

# CONTROL – TOTAL

. summarize totaldiff

Variable |	Obs	Mean	Std. Dev. Min	Max

-------------+--------------------------------------------------------

totaldiff |	210	14.65714	5.413661	6	35

# TOKYO (CONTROL) – ELEMENTARY

. summarize totaldiff

Variable |	Obs	Mean	Std. Dev. Min	Max

-------------+--------------------------------------------------------

totaldiff |	75	15.38667	5.67806	7	30

# NAGANO (CONTROL) - MIDDLE

. summarize totaldiff

Variable |	Obs	Mean	Std. Dev. Min	Max

-------------+--------------------------------------------------------

totaldiff |	135	14.25185	5.23866	6	35

# T-TEST COMPARING CONTROL WITH FUKUSHIMA

. ttesti 210 14.65714 5.413661 322 15.63665 6.165043

Two-sample t test with equal variances

------------------------------------------------------------------------------

| Obs Mean Std. Err. Std. Dev. [95% Conf. Interval]

---------+--------------------------------------------------------------------

x |	210	14.65714	.3735781	5.413661	13.92068	15.3936

y |	322	15.63665	.3435644	6.165043	14.96073	16.31257

---------+--------------------------------------------------------------------

combined |	532	15.25	.2555458	5.894195	14.748	15.75201

---------+--------------------------------------------------------------------

diff |	-.97951	.5215691	-2.004106	.0450863

------------------------------------------------------------------------------

diff = mean(x) - mean(y)	t = -1.8780

Ho: diff = 0	degrees of freedom =	530

Ha: diff < 0	Ha: diff != 0	Ha: diff > 0

Pr(T < t) = 0.0305	Pr(|T| > |t|) = 0.0609	Pr(T > t) = 0.9695

# T-TEST COMPARING CONTROL WITH RELOCATED

ttesti 210 14.65714 5.413661 50 17.22 6.987715

Two-sample t test with equal variances

------------------------------------------------------------------------------

| Obs Mean Std. Err. Std. Dev. [95% Conf. Interval]

---------+--------------------------------------------------------------------

x |	210	14.65714	.3735781	5.413661	13.92068	15.3936

y |	50	17.22	.9882121	6.987715	15.23411	19.20589

---------+--------------------------------------------------------------------

combined |	260	15.15	.3611508	5.823382	14.43883	15.86116

---------+--------------------------------------------------------------------

diff |	-2.56286	.9041663	-4.343345	-.7823745

------------------------------------------------------------------------------

diff = mean(x) - mean(y)	t = -2.8345

Ho: diff = 0	degrees of freedom =	258

Ha: diff < 0	Ha: diff != 0	Ha: diff > 0

Pr(T < t) = 0.0025	Pr(|T| > |t|) = 0.0050	Pr(T > t) = 0.9975

# T-TEST COMPARING FUKUSHIMA WITH RELOCATED

. ttesti 322 15.63665 6.165043 50 17.22 6.987715

Two-sample t test with equal variances

------------------------------------------------------------------------------

| Obs Mean Std. Err. Std. Dev. [95% Conf. Interval]

---------+--------------------------------------------------------------------

x |	322	15.63665	.3435644	6.165043	14.96073	16.31257

y |	50	17.22	.9882121	6.987715	15.23411	19.20589

---------+--------------------------------------------------------------------

combined |	372	15.84947	.3263799	6.294988	15.20768	16.49125

---------+--------------------------------------------------------------------

diff |	-1.58335	.954621	-3.460513	.2938131

------------------------------------------------------------------------------

diff = mean(x) - mean(y)	t = -1.6586

Ho: diff = 0	degrees of freedom =	370

Ha: diff < 0	Ha: diff != 0	Ha: diff > 0

Pr(T < t) = 0.0490	Pr(|T| > |t|) = 0.0980	Pr(T > t) = 0.9510

**ONE-WAY ANOVA BETWEEN THREE GROUPS**

. oneway totaldiff location

Analysis of Variance

Source	SS	df	MS	F	Prob > F

------------------------------------------------------------------------

Between groups	267.111012	2	133.555506	3.69	0.0256

Within groups	20931.1059	578	36.2129859

------------------------------------------------------------------------

Total	21198.2169	580	36.5486498

Bartlett's test for equal variances: chi2(2) =	5.7089 Prob>chi2 = 0.058

**KRUSKILL-WALLIS BETWEEN THREE GROUPS**

. kwallis totaldiff, by(location)

Kruskal-Wallis equality-of-populations rank test

+---------------------------+

| location | Obs | Rank Sum |

|----------+-----+----------|

| Relocated | 50 | 16633.00 |

| Native | 322 | 95143.00 |

| Control | 209 | 57295.00 |

+---------------------------+

chi-squared =	5.417 with 2 d.f.

probability =	0.0666

chi-squared with ties =	5.436 with 2 d.f.

probability =	0.0660

.
