# Supplementary material for: Assessing the Mental Health Impact of the 2011 Great Japan Earthquake, Tsunami, and Radiation Disaster on Elementary and Middle School Children in the Fukushima Prefecture of Japan
Source: PLoS One. 2017 Jan 18;12(1):e0170402. doi: 10.1371/journal.pone.0170402 (PMC5242473; doi:10.1371/journal.pone.0170402)
Supplement: S1 Supporting Information — (ZIP) [file pone.0170402.s001.zip › Demographics Koriyama.doc]

```
2012 Earthquake/Tsunami/Radiation Disaster Questionnaire

First is a question regarding the relationship between the individual filling out this survey and the child. From the child’s perspective, please circle the role of the individual filling out this survey.
１　Mother　　２　Father　　３　Grandmother　　４　Grandfather　　５Other（　　　　　）
Questions regarding the child.
Question１　Please circle the child’s gender. 　　　　　　	１　Boy	　２　Girl
Question ２　Please write the child’s year and month of birth. 　　　 Year 　　　 Month
Question３　Who is the child currently living with. Please circle all that apply. If there are siblings, please write their age.
１　Mother        ２　Father　　　　　３　Siblings（　　）How many
４　Maternal Grandpa５　Maternal Grandma６　Paternal Grandpa７　Paternal Grandma
８　Maternal aunt/uncle（　　）How many　　　　　９　Paternal aunt/uncle（　　）How many
１０　Other（　　）How many
（Supplemental Question）Siblings’ age（If more than 3, please write in the margin）
Sibling １	　１　Boy　　２　Girl　　　　　　years old
Sibling ２	　１　Boy　　２　Girl　　　　　　years old
Sibling ３	　１　Boy　　２　Girl　　　　　　years old

Question ４　After the Great East Japan Earthquake, did the child move or have damage to the home? Please circle the relevant choice.
１　Did not move or have damage to home
２　Moved due to that earthquake
３　Had damage to the home due to that earthquake
（Supplemental question）If the child moved, how many times did the child move?
１　１time　　２　２times　　３　３times　　４　４times　　５　５and more times

Question５　What housing does the child live in now? Please circle the relevant choice.
１　Free standing house　　２　Luxury apartment　　３　Apartment
４　Relative’s house ５　Temporary housing　　　　６　Friend’s house
７　Other（　　　　　）

Question６　Please write where the child’s current lives.
　　　　　Prefecture　　　　　City　　　　　Town

（Supplemental Question） After the Great East Japan Earthquake, if the child moved, please write where the child lived before the disaster.
　　　　　Prefecture　　　　　City　　　　　Town
【Regarding the Mother and Father】

Question７　Questions regarding before the Great East Japan Earthquake (February 2011) and now. Please circle the relevant choice.
```

| 【Mother】 | | | 【Father】 | |
| --- | --- | --- | --- | --- |
| Before Great East Japan Disaster | Now | Before Great East Japan Disaster | | Now |
| １　Unemployed  ２　Student  ３　Employed full-time  ４　Employed part-time  ５　Self-employed/ Family business  ６　Side job  ７　Other（　　　　　　　　） | １　Unemployed  ２　Student  ３　Employed full-time  ４　Employed part-time  ５　Self-employed/ Family business  ６　Side job  ７　Other（　　　　　　　　） | １　Unemployed  ２　Student  ３　Employed full-time  ４　Employed part-time５　Self-employed/ Family business  ６　Side job  ７　Other（　　　　　　　　） | | １　Unemployed  ２　Student  ３　Employed full-time  ４　Employed part-time  ５　Self-employed/ Family business  ６　Side job  ７　Other（　　　　　　　　） |

```
（Supplemental Question）Is the father a bachelor。Please circle the relevant choice.
　１　Yes　　２　No

Question８ Question about income and hours worked after the Great East Japan Earthquake. Please circle the relevant choice.
```

| 【Mother】 | | | 【Father】 | |
| --- | --- | --- | --- | --- |
| Hours worked | Income | Hours worked | | Income |
| １　No change  ２　Increased  ３　Decreased  ４　Other（　　　　　　　　） | １　No change  ２　Increased  ３　Decreased  ４　Other（　　　　　　　　） | １　No change  ２　Increased  ３　Decreased  ４　Other（　　　　　　　　） | | １　No change  ２　Increased  ３　Decreased  ４　Other（　　　　　　　　） |

Question９　Question about the highest level that the child’s parents graduated from. Please circle the relevant choice.

| 【Mother】 | 【Father】 |
| --- | --- |
| １　Middle school  ２　Vocational school（After graduating middle school）  ３　High school  ４　Vocational school（After graduating high school）  ５　Junior college  ６　College  ７　Graduate school  ８　Other（　　　　　　　　） | １　Middle school  ２　Vocational school (After graduating middle school）  ３　High school  ４　Vocational s(After graduating high school）  ５　Junior college  ６　College  ７　Graduate school  ８　Other（　　　　　　　　） |

```
【Regarding the child】

Question１０　Question regarding the child’s education. Please circle the relevant choice. 　After the disaster, did the child change schools? 　１　Yes（　　　times）　　２　No
Question１１　With whom did the child live with before the Great East Japan Earthquake? Please circle all relevant choices.
１　Mother ２　Father　　　　　３　Siblings（　　）How many
４　Maternal Grandpa５　Maternal Grandma６　Paternal Grandpa７　Paternal Grandma
８　Maternal aunt/uncle（　　）How many　　　　　９　Paternal aunt/uncle（　　）How many
１０　Other（　　）How many
Question regarding the child’s experience during the Great East Japan Earthquake. Please circle the relevant choice.
Question１２　During the earthquake and tsunami, where was the child?
１　School　　２　Home　　３　Other（　　　　　　　　）

Question１３　Questions regarding the child’s experiences during the earthquake. Please circle the relevant choice.
１　The child got hurt him/herself.
２　Family member or friend got hurt.
Who was injured? Please circle all relevant choices.
　　　ア　Mother　　　　イ　Father　　　　　ウ　Sibling(s)
　　　エ　Maternal grandpa オ　Maternal grandma　　カ　Paternal grandpa キ　Paternal grandma
ク　Maternal aunt/uncle ケ　Paternal aunt/uncle
コ　他の新成　　　　　サ　Other adults シ　Child’s friend(s)
３　Family member or friend died.
Who died? Please circle all relevant choices.
　　　ア　Mother　　　　　イ　Father　　　　　ウ　Sibling(s)
　　　エ　Maternal Grandpaオ　Maternal Grandmaカ　Paternal Grandpa　　キ　Paternal Grandma
ク　Maternal aunt/uncle　　　　　　　　　　ケ　Paternal aunt/uncle
コ　Other relative　　　　　サ　Other adult　　　　　シ　Child’s friend(s)
Question１４　Regarding the child’s experience during the earthquake, please circle the relevant choice.
１　During the earthquake, the child saw someone get hurt
２　During the earthquake, the child saw someone die
３　During the earthquake, the child tried to help rescue someone
４　A relative tried to help rescue someone

Question１５　Question regarding the child’s pet. Please circle the relevant choice.
１  The child does not own a pet.
２　The pet was injured during the disaster.
３　The pet died during the disaster.
４　The pet became lost during the disaster.
５　The pet was left behind during relocation.
６　The pet is currently living with the child.
Question１６　Question regarding the child’s residence during and after the disaster. Please circle all relevant choices.
１　No evacuation was necessary
２　Evacuation was necessary and at that time, the child left behind clothes or toys
４　The child’s home was lost
５　The child saw neighborhood houses or buildings damaged or destroyed
６　The child saw other houses or buildings damaged or destroyed
７　The child lived for a short time away from the parent or caregiver after the earthquake
８　The child lived for a long time away from the parent or caregiver after the earthquake
９　The child stayed with people who the child does not usually live with after the earthquake
【Radiation Risk】
Below are questions regarding the radiation risk and effects. Please circle the relevant choice.
```

**8. Please write freely about anything：**

|  | Not worried at all | Not worried | A little worried | Worried | Extremely worried |
| --- | --- | --- | --- | --- | --- |
| 1. How worried are you about radiation contaminating food | １ | ２ | ３ | ４ | ５ |
| 2. How worried are you about radiation contaminating drinks | １ | ２ | ３ | ４ | ５ |
| 3. How worried are you about a family member becoming ill due to radiation in the next 5 years | １ | ２ | ３ | ４ | ５ |
|  | Not thinking about at all | Not thinking about | Thinking about a little | Thinking about | Seriously thinking about |
| 4. Are you thinking about relocating further away within 5 years? | １ | ２ | ３ | ４ | ５ |
|  | Not feeling at all | Not feeling | Feeling a little | Feeling | Seriously feeling |
| 5. Are you feeling that necessary information regarding the radiation is easy to obtain? | １ | ２ | ３ | ４ | ５ |
|  | Extremely decreased | Decreased | No change | Increased | Extremely increased |
| 6.How is your worry compared to initially after the disaster | １ | ２ | ３ | ４ | ５ |
|  | Absolutely insufficient | Insufficient | Sufficient | Plentiful | Extremely plentiful |
| 7. How do you feel about measures taken to protect children or pregnant women | １ | ２ | ３ | ４ | ５ |

**Thank you for participating in this survey.**
